# The lipidome, genotoxicity, hematotoxicity and antioxidant properties of andiroba oil from the Brazilian Amazon

**DOI:** 10.1590/1678-4685-GMB-2015-0098

**Published:** 2016-05-13

**Authors:** Susana Suely Rodrigues Milhomem-Paixão, Maria Luiza Fascineli, Mariana Matos Roll, João Paulo Figueiró Longo, Ricardo Bentes Azevedo, Julio Cesar Pieczarka, Hugo Leonardo Crisóstomo Salgado, Alberdan Silva Santos, Cesar Koppe Grisolia

**Affiliations:** 1 Laboratório de Citogenética, Instituto de Ciências Biológicas, Universidade Federal do Pará (UFPA), Belém, PA, Brazil; 2 Laboratório de Nanobiotecnologia, Departamento de Genética e Morfologia, Instituto de Ciências Biológicas, Universidade de Brasília (UnB), Brasília, DF, Brazil; 3 Laboratório de Genética Toxicológica, Departamento de Genética e Morfologia, Instituto de Ciências Biológicas, Universidade de Brasília (UnB), Brasília, DF, Brazil; 4 Laboratórios de Investigação Sistemática em Biotecnologia e Biodiversidade Molecular (LabISisBio), Instituto de Ciências Exatas e Naturais, Universidade Federal do Pará (UFPA), Belém, PA, Brazil

**Keywords:** acute toxicity, antioxidant activity, Carapa guianensis, genotoxicity, lipidomics

## Abstract

Andirobeira is an Amazonian tree, the seeds of which produce a commercially valuable oil that is used in folk medicine and in the cosmetic industry. Andiroba oil contains components with anti-inflammatory, cicatrizing and insect-repellant actions. However, virtually nothing is known of the safety of this oil for humans. The aim of this work was therefore to investigate the hematotoxicity, genotoxicity and mutagenicity of andiroba oil using the comet and micronucleus assays, and to assess its antioxidant properties and lipidome as a means of addressing safety issues. For the experiments, andiroba oil was administered by gavage for 14 consecutive days in nulliparous female Swiss mice randomly distributed in four groups: negative control and three doses of oil (500, 1000 and 2000 mg/kg/day). These doses were chosen based on recommendations of the OECD guideline no. 474 (1997). GC/MS was used to investigate the free fatty acid, cholesterol and triterpene content of andiroba oil in a lipidomic analysis. No clinical or behavioral alterations were observed throughout the period of treatment, and exposure to andiroba oil at the doses and conditions used here did not result in hematotoxic, genotoxic or mutagenic effects. Tests *in vitro* showed that oil sample 3 from southwestern of Brazilian Amazon had a high antioxidant capacity that may protect biological systems from oxidative stress, although this activity remains to be demonstrated *in vivo*.

## Introduction

Andiroba oil is extracted from the seeds of the andiroba tree, represented by two species, *Carapa guianensis* and *Carapa procera*, of the family Meliaceae that occurs in the Amazon (Fisch *et al.*, 1995). This oil has various uses in folk medicine, such as a cicatrizing and anti-inflammatory agent and as an insect repellent (Bauch and Dunisch, 2000; Ferraz *et al.*, 2002; Shanley *et al.*, 2005); in recent years, andiroba oil has also been used intensively in the cosmetic industry (Ferreira *et al.*, 2010).

Previous studies have reported acaricidal (Farias *et al.*, 2007, 2009), larvicidal (Silva *et al.*, 2004), insect repellent (Miot *et al.*, 2004, 2011) and antiplasmodial (Miranda Júnior CRN, 2010, MSc dissertation, Universidade Federal do Pará, Belém, PA, Brazil) activities for andiroba oil. However, a general lack of knowledge regarding the toxicity and/or proof of pharmacological activities of natural products employed as medicines and their indiscriminate use may put Amazon populations at risk of adverse reactions (Frescura VDA, 2012, MSc dissertation, Universidade Federal de Santa Maria, Santa Maria, RS, Brazil).

Andiroba oil is rich in essential fatty acids, including oleic, palmitic, stearic and linoleic acids (Martinborough, 2002; Rain-Tree, 2014). Linoleic acid reduces cholesterol levels and blood pressure and is beneficial in the prevention of cancer. Andiroba oil also contains meliacines, a group of substances that confer a bitter taste to the oil and have antimalarial and antiparasitic activities; there are also limonoids with anti-inflammatory, insect repellent and anti-tumoral properties (Martinborough, 2002; Rain-Tree, 2014).

Stearic, palmitic, oleic and linoleic fatty acids can cause apoptosis in different cell lines *in vitro*, depending on the concentration tested (Mu *et al.*, 2001; Lu *et al.*, 2003; Cury-Boaventura *et al.*, 2004). There has been much discussion about the possible mechanisms involved in the induction of apoptosis by these acids and the physiological effects that they produce (Curi *et al.*, 2002). One of the best characterized mechanisms in apoptosis involves damage to cellular DNA that may occur via various routes (Batista LFZ, 2008, PhD thesis, Universidade de São Paulo, São Paulo, SP, Brazil). This damage can be eliminated by the endogenous DNA repair system of cells, but if the damage persists it may induce genotoxicity, mutagenesis or even apoptosis (Wong *et al.*, 2005; Asare *et al.*, 2011; Batista LFZ, 2008, PhD thesis, Universidade de São Paulo, São Paulo, SP, Brazil).

Knowledge about the genotoxic potential of natural compounds and semi-synthetic and synthetic chemicals is essential for regulatory agencies so that they can establish the risk to humans (Ribeiro *et al.*, 2003). To date, few studies have examined the toxicity of andiroba oil and there are no reports on the possible genotoxic or mutagenic effects of this oil. The aim of this work was therefore to investigate the possible toxicological, mutagenic and genotoxic effects of andiroba oil in Swiss mice and to examine the antioxidant properties of this oil *in vitro*. The data obtained contribute to our understanding of the safety of this oil and its saponified fraction.

## Material and Methods

### Plant material

Seeds from andiroba trees were collected from 21 sites in the state of Pará in the Brazilian Amazon under license no. MMA/ICMBIO/SISBIO-33336-1 issued by the Brazilian Environmental Agency. The sample identifications, mesoregions, municipalities, geographical coordinates and collection periods for these seeds are described in Table 1. The exsicates were deposited in the herbarium of the Brazilian Agricultural Research Company (EMBRAPA) under accession number 191736. The seeds were identified by Dr. Regina Celia Viana Martins da Silva, curator of the IAN Embrapa Amazônia Oriental herbarium.

**Table 1:** Samples, localities and periods of collection of andiroba seeds and the concentration of oil causing a 50% reduction in the DPPH radical (EC_50_).

Samples	Mesoregion	Municipality	Geographical coordinates	Collection period	EC_50_ (μL/mL)
SE1	Southeast	Nova Ipixuna	S 04° 48′ 30.1″ W 049° 21′ 42.8″	16-20/4/12	86.62
SE2	Jacundá	S 04° 27′ 03.0″ W 049° 06′ 59.5″	16-20/4/12	151.0	
SE3	São Miguel do Guamá	S 01° 35′ 28.5″ W 047° 34′ 39.5″	3-7/5/2012	126.7	
NE1	Northeast	Abaetetuba	S 01° 43′ 04″ W 048° 52′ 58″	3-7/5/2012	2305
NE2	Acará	S 01° 58′ 25.9″ W 048° 18′ 49.5″	3-7/5/2012	302.0	
NE3	Cametá	S 02° 14′ 40.0″ W 049° 29′ 45.0″	3-7/5/2012	204.3	
LAM1	Lower Amazon	Santarém	S 02° 24′ 52″ W 054° 42′ 36.0″	25-29/3/2012	43.56
LAM2	Oriximiná	S 01° 30′ 55.3″ W 055° 46′ 52.0″	25-29/3/2012	150.7	
LAM3	Óbidos	S 01° 39′ 21.5″ W 055° 37′ 15.9″	25-29/3/2012	45.20	
MAR1	Marajó	Curralinho	S 01° 45′ 59.1″ W 049° 49′ 45.2″	12/4; 23/5 and 24/7/2012	468.9
MAR2	Salvaterra	S 00° 48′ 02.0″ W 048° 32′ 01.0″	12/4; 23/5 and 24/7/2012	90.23	
MAR3	Breves	S 01° 38′ 17.9″ W 050° 27′ 99.5″	12/4; 23/5 and 24/7/2012	1894	
MTR1	Metropolitan	Santa Isabel do Pará	S 01° 21′ 12.4″ W 048° 08′ 37.3″	29/3-1/4/2012	326.3
MTR2	Castanhal	S 01° 17′ 49.5″ W 047° 55′ 19.7″	29/3-1/4/2012	559.3	
MTR3	Bujarú	S 01° 35′ 46.3″ W 047° 57′ 32.9″	29/3-1/4/2012	252.6	
SW1	Southwest	Porto de Moz	S 01° 45′ 00″ W 052° 14′ 15.0″	25-29/3/2012	425.4
SW2	Medicilândia	S 03° 23′ 59.0″ W 052° 53′ 36.8″	12/3-24/4/2012	1181	
SW3	Uruará	S 03° 58′ 31.7″ W 053° 37′ 32.1″	12/3-24/4/2012	59.95	
SW4	Itaituba	S 04° 16′ 34.0″ W 055° 59′ 01.0″	29/3-5/4/2012	150.0	
SW5	Jacareacanga	S 06° 13′ 20.0″ W 057° 45′ 10.0″	29/3-5/4/2012	100.4	
SW6	Aveiro	S 03° 50′ 30.0″ W 055° 28′ 32.7″	29/3-5/4/2012	106.7	
IANA*					8913
AMAZON*					204.8

S – South, W – West. EC_50_: minimum concentration required to reduce 50% of DPPH.

* Commercial samples: Amazon Ervas and Iana^®^ D'amazônia.

### Collection of andiroba oil

Andiroba oil from *C. guianensis* from each site was processed and characterized separately in the Laboratory for Systematic Investigation in Biotechnology and Fine Chemistry (LabISisBio) at the Federal University of Pará. After collection, the fruits were stored at 12 °C and transported to the lab where the oil was extracted either with an artisanal extraction process (Shanley *et al.*, 2005) or *in natura* using organic solvent (Sousa Filho JVC, 2007, MSc dissertation, Universidade Federal do Pará, Belém, PA, Brazil). After artisanal extraction, the resulting mass was pressed and squeezed to reduce the oil content to < 5%, as described elsewhere (Gunstone and Padley, 1997). After extraction in organic solvent, the solvent was removed by evaporation to obtain oil without hexane that could be fractionated and separated. The derivatization technique to characterize the intact oil (sample SW3) and saponified and unsaponified compounds used in this study was essentially that described by Souza *et al.* (2014), with some modifications in the temperature programming and column change.

### Antioxidant assay

To choose the sample that would be used in this study, the antioxidant capacity of the samples was evaluated by the 2,2-diphenyl-1-picryl-hydrazyl (DPPH) method. The protocol followed was that described by Brand-Williams *et al.* (1995) with adaptations described by Razali *et al.* (2008) and Atmani *et al.* (2009) in which 900 μL of DPPH solution (0.06 mM) was mixed with 100 μL of oil diluted in methanol (12.5, 25, 50, 100 to 200 μL/mL) followed by incubation for 20 min. The absorbance was read in a Spectramax M2 spectrophotometer (Molecular Devices, Sunnyvale, CA, USA) at 515 nm. The results were expressed as the percentage inhibition of the DPPH radical that was calculated using the absorbances measured above in conjunction with the following formula:

$$\%\;\text{inhibition}=\frac{\left[A0-\left(A1-AS\right)\right]}{A0}\times 100$$

where *A0* is the absorbance of DPPH, *A1* is the absorbance of DPPH + diluted oils and *As* is the absorbance of diluted oils. The percentages of inhibition were used to calculate the EC_50_ (effective concentration or minimum concentration required to reduce the DPPH radical by 50%) by non-linear regression. Based on the results of the DPPH assay, the sample with the greatest antioxidant potential (sample SW3) and that was available in sufficient quantity was selected for the *in vivo* tests.

### Experimental design

For the *in vivo* assays, 90-day-old non-pregnant nulliparous female Swiss mice (*Mus musculus*) purchased from the Multidisciplinay Center for Biological Investigation (CEMIB) at the State University of Campinas (UNICAMP), Campinas, SP, Brazil, were used. The mice were maintained in the animal house of the Department of Genetics and Morphology at the University of Brasília under the following conditions: 23 ± 2 °C, relative humidity 30-70%, and a 12 h light/dark cycle, with 10-15 changes of air/h. The mice had access to drinking water and commercial rodent chow *ad libitum*. The animal protocols were approved by the Committee for Ethics in Animal Use (CEUA) of the Institute of Biological Sciences at the University of Brasília (protocol no. 127331/2013).

The mice were randomly allocated to one of four experimental groups (n = 6 mice/group): negative control and three doses of oil (500, 1000 and 2000 mg/kg/day). The negative control group received corn oil (Salada^®^, Bunge) and the treated mice received a solution of andiroba oil and corn oil by gavage through a gastric tube. The treatments were done for 14 consecutive days and the dose limit corresponded to that recommended in the Organization for Economic Co-operation and Development (OECD) guidelines for the evaluation of genotoxicity by the micronucleus test (Macgregor *et al.*, 1987) and of chromosomal aberrations in bone marrow (Preston *et al.*, 1987). Throughout the experimental period, the mice were weighed on days 0, 3, 6, 9, 12 and 15, and food consumption was monitored on days 3, 6, 9, 12 and 15. All of the mice were examined daily throughout the treatment period to check for possible clinical alterations/symptoms.

On the 15^th^ day the mice were sedated with a mixture of ketamine chlorohydrate (10%) and xylazine (2%) and, after confirmation of narcosis, blood samples were obtained. Blood was collected by cardiac puncture and stored in plastic microtubes with 10% EDTA for hematological evaluation and preparation of slides for the comet assay. During autopsy, the mice were inspected for macroscopic alterations and the liver, kidneys and spleen were collected and weighed. For the micronucleus and nuclear abnormality tests, slides were prepared using femur bone marrow.

### Comet assay

The protocol for this assay was based on the alkaline comet assay (pH > 13) described by Singh *et al.* (1988), with some modifications. The slides were analyzed in a Zeiss-Axioskop 2 fluorescence microscope fitted with a 510-560 nm filter, a 590 nm blocking filter and a magnification of 400x. Ethidium bromide (20 μg/mL) was used for staining. Nucleoids were evaluated based on their level of fragmentation that was scored from 0 to 4 (Collins, 2004). For each mouse, 100 cells were evaluated and classified based on the damage caused by the tested substance and these data were then used to calculate the damage index (DI). The DI was defined as the product obtained by multiplying the number of comets from each class with the digit denominating the class (0, 1, 2, 3 and 4), the formula being taken from Jaloszynski *et al.* (1997).

### Micronucleus test

The micronucleus test was done using mouse femur bone marrow, as described by Schmid (1975), with modifications. The stained slides were examined with an Olympus BH2 light microscope at a magnification of 1000x. For each mouse, 4000 erythrocytes were counted, of which 2000 were polychromatic (PCE – polychromatic erythrocytes) and 2000 were normochromatic (NCE – normochromatic erythrocytes). When the cell count reached 2000, the values for the two cell populations were recorded in order to assess the cytotoxicity and calculate the PCE/NCE ratio. The lower this ratio, the greater the cytotoxicity involving a significant reduction in PCE. In addition to this ratio, the appearance of the micronucleated polychromatic cells was also recorded.

### Hematological analysis

For hematological analysis, 370 μL of blood containing 10% disodium EDTA as anticoagulant was analyzed in an automatic veterinary hematocytometer (Sysmex pocH 100iV Diff™) calibrated for mice.

### Gas chromatography-mass spectrometry (GC-MS) analysis and compound identification

GC-MS analysis was done with a Thermo GC/MS system equipped with a quadrupole mass selective detector operated at 70eV in electron impact (EI) mode. The TRACE-1300 gas chromatograph (GC) was equipped with a RTX-5TG- RESTECK column (30 m x 0.25 mm di x 0.25 μm film thickness and the oven was operated at 40-300 °C in increments of 10 °C/min with He as the carrier gas (flow: 1 mL/min). The injector was set at 220 °C and 1 μL of the sample was injected into the GC via an AI/AS-1310 autosampler. The mass spectrometer (MS) was operated in scan mode (start after 3 min, mass range from 40-800 a.m.u. at 1 scan/s). The transfer line and ion source were both operated at 280 °C. The compounds were identified by comparing their mass spectra with those of the NIST-11MS lipid library database.

### Statistical analysis

The EC_50_ (effective concentration capable of inhibiting 50% of the free radical) was calculated by non-linear regression from the percentage inhibition of the free radical in the DPPH assay for the 21 samples of andiroba oil. The quantitative data were evaluated by parametric or non-parametric statistical methods based on the normal distribution of the data. Parametric data were compared by analysis of variance (ANOVA) followed by Dunnett's test, whereas non-parametric data were analyzed with the Wilcoxon text followed by the Kruskall-Wallis test. All data comparisons and statistical analyses were done using the softwares Instat 3.02 and Prism 5.0 (GraphPad Inc., La Jolla, CA, USA), with a value of p ≤ 0.05 indicating significance.

## Results

All of the oil samples showed antioxidant activity towards the DPPH radical, as shown in Table 1, which summarizes the EC_50_ data. Supplementary Figures S1-S7 show that there was a correlation between the percentage of DPPH inhibition and the oil concentration needed to cause a 50% reduction of DPPH. Samples with high and low antioxidant properties showed regional variations, *e.g.*, northeastern Pará State, Marajó island and the metropolitan region of Belém, as well as variations within each region. Two commercial samples of andiroba oil (Amazon Ervas and Iana^®^ D'amazônia) were also tested for their antioxidant potential. The Iana sample, diluted in mineral oil as stated on the manufacturer's label, had the lowest antioxidant capacity of all the samples tested, with an EC_50_ of 8913 μL/mL, compared to 204.8 μL/mL for the Amazon Ervas sample, which was similar to the value for the Cametá sample from northeastern Brazil.

Based on these results and the availability of each oil, the sample SW3 was chosen for biological assays. The lipid profiles were determined using the derivatization technique. Saponified compounds represented > 97% of the sample and non-saponified compounds consisted of steroids and triterpenoids derivatives (Table 2). More than 97% of the fatty acids were represented by oleic, palmitic, stearic, linoleic and arachidonic acids. However, the derivatization technique was also capable of detecting traces of other acids in the same sample. These findings agreed with those reported for other analyses of andiroba oil (Martinborough, 2002).

**Table 2:** Profile of fatty acids, steroids and triterpenes present in two samples (SW3-1 and SW3-2) of andiroba oil.

Component	Peak retention time (min)	Retention index	Oil profile	SW3-1	SW3-2
		Lipids 1 (%)	Lipids 2 (%)		
Fatty acids	15.52	1899	Miristic acid	0.07 ± 0.03	0.13 ± 0.02
	17.54	2027	Palmitic acid	37.95 ± 0.63	32.21 ± 0.94
	17.86	2048	Palmitoleic acid	0.07 ± 0.12	0.81 ± 0.07
	20.49	2234	Oleic acid	55.23 ± 0.43	41.92 ± 0.52
	21.37	2301	Linoleic acid	4.21 ± 0.34	7.63 ± 0.36
	21.58	2318	Stearic acid	0.03 ± 0.06	14.53 ± 0.75
	22.93	2428	Arachidic acid	1.07 ± 0.19	2.12 ± 0.39
	25.52	2664	Behenic acid	0.28 ± 0.07	0.43 ± 0.06
	27.73	2894	Lignoceric acid	0.17 ± 0.06	0.25 ± 0.01
					
Steroids	28.59	Ns	Squalene	0.43 ± 0.61	-
	30.86	Ns	Stigmasterol	0.11 ± 0.04	-
	31.87	Ns	Cholesterol	0.07 ± 0.02	-
					
Triterpenes	33.11	Ns	Epoxygedunnin*	0.08 ± 0.02	-
	33.64	Ns	1,3-Dipalmitin	0.09 ± 0.02	-
	33.94	Ns	Deoxylactone-derivative	0.13 ± 0.02	-
	35.92	Ns	Deacetylgedunin*	0.04 ± 0.01	-
	36.40	Ns	Epoxydeacetylgedunin*	0.06 ± 0.03	-

Ns = not simulated (requires hydrocarbons with a carbon number > 30),

* Tentative identification.

Lipids 1 = fermented seeds (extracted by oil dripping). Lipids 2 = non-fermented seeds (extracted with solvent). The values are the mean ± SD of chromatographic quantifications. Retention Index: relative retention-time of a compound within the chromatograph according to its chromatographic properties. SW3-1 and SW3-2 means two chromatographic analysis of same sample.

To evaluate the possible toxicity of SW3 *in vivo*, the mice were observed daily for any alterations during treatment, but no clinical or behavioral alterations were observed and there were no changes in body weight. No macroscopic alterations were seen during autopsy nor were there any alterations in the absolute or relative weight of the kidneys, liver or spleen (Table 3).

**Table 3:** Absolute and relative weights of mouse organs after treatment with andiroba oil.

		Andiroba oil (mg/kg/day)		
	Negative control	500	1000	2000
Absolute weight				
Liver	1.82 ± 0.29	1.96 ± 0.31	1.86 ± 0.17	2.16 ± 0.30
Spleen	0.15 ± 0.08	0.15 ± 0.02	0.13 ± 0.01	0.13 ± 0.02
Right kidney	0.17 ± 0.02	0.18 ± 0.02	0.17 ± 0.02	0.20 ± 0.02
Left kidney	0.18 ± 0.02	0.18 ± 0.02	0.17 ± 0.01	0.19 ± 0.02
				
Relative weight				
Liver	4.81 ± 0.19	5.10 ± 0.35	5.26 ± 0.51	5.29 ± 0.45
Spleen	0.39 ± 0.16	0.40 ± 0.08	± 0.08	0.33 ± 0.05
Right kidney	0.46 ± 0.05	0.48 ± 0.03	0.48 ± 0.05	0.49 ± 0.05
Left kidney	± 0.05	0.47 ± 0.04	0.48 ± 0.05	0.48 ± 0.06

The relative weight is expressed in relation to the final body weight of each animal. The values are the mean ± SD (n = 6 animals/group) and were analyzed by ANOVA or the Kruskal-Wallis test, depending on the normality of the data distribution. There were no significant differences between the responses to andiroba oil and the negative control, or among the doses of oil tested.

**Figure 1:**
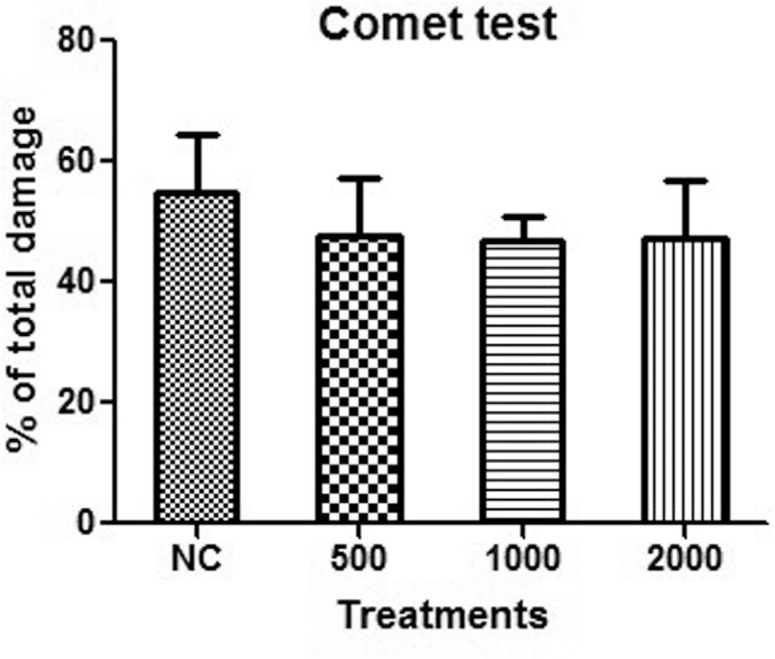
Comet test. The doses of andiroba oil are in mg/kg/day. NC – negative control. The columns represent the mean ± SD and were analyzed by ANOVA. There were no significant differences between the responses to andiroba oil and the negative control, or among the doses of oil tested.

The comet assay (Figure 1) and micronucleus test (Table 4) were used to assess the genotoxicity of SW3. No significant alterations were observed in micronucleus formation or DNA fragmentation during treatment with andiroba oil. There were also no hematological alterations in the mice or nuclear abnormalities in blood cells (polymorphonuclear or normonuclear cells) (Table 5).

**Table 4:** Frequency of micronuclei after treatment with andiroba oil.

		Andiroba oil (mg/kg/day)		
Parameter	Negative control	500	1000	2000
MN-PCE (number)	1.66 ± 2.42	2 ± 2.09	1.16 ± 1.60	2.5 ± 1.22
MN-PCE (%)	8.33 ± 12.11	10.00 ± 10.49	5.83 ± 8.01	12.50 ± 6.12
PCE/NCE ratio	1.77 ± 0.57	2.39 ± 0.60	2.10 ± 0.63	2.29 ± 0.42

MN – micronuclei, NCE – normochromatic erythrocytes, PCE – polychromatic erythrocytes. The values represent the mean ± SD and were analyzed by ANOVA. There were no significant differences between the responses to andiroba oil and the negative control, or among the doses of oil tested.

**Table 5:** Erythrocyte, leukocyte and platelet counts and characteristics after treatment with andiroba oil.

		Andiroba oil (mg/kg/day)		
Parameters	Negative control	500	1000	2000
Erythrocytes				
RBC x 10^6^/μL	8.80 ± 0.35	8.93 ± 0.40	9.04 ± 0.29	8.57 ± 0.38
HGB (g/dL)	12.48 ± 0.45	12.50 ± 0.41	12.65 ± 0.40	12.07 ± 0.42
HCT (%)	32.23 ± 1.22	32.58 ± 1.12	32.75 ± 0.91	31.38 ± 1.05
MCV (fL)	36.7 ± 0.69	36.5 ± 0.72	36.2 ± 0.53	36.6 ± 0.64
MCH (pg)	14.2 ± 0.3	14.0 ± 0.5	14.0 ± 0.2	14.1 ± 0.2
MCHC (g/dL)	38.7 ± 0.41	38.4 ± 0.56	38.6 ± 0.48	38.5 ± 0.31
RDW (%)	13.57 ± 0.69	14.37 ± 1.13	14.17 ± 0.83	13.62 ± 0.78
Leukocytes				
WBC x 10^3^/μL	2.23 ± 1.12	2.32 ± 1.16	2.53 ± 1.13	1.78 ± 0.75
W-SCR (%)	71.57 ± 9.24	71.45 ± 16.52	75.95 ± 10.58	76.38 ± 9.21
W-MCR (%)	27.78 ± 8.41	28.00 ± 16.16	23.57 ± 10.27	22.38 ± 7.28
W-LCR (%)	0.65 ± 1.02	0.38 ± 0.52	0.48 ± 0.68	1.23 ± 2.88
Platelets				
PLT x 10^3^/mL	1371 ± 207	1479 ± 248	1442 ± 231	1340 ± 295
PDW (fL)	7.02 ± 0.48	7.00 ± 0.20	6.80 ± 0.11	7.00 ± 0.17
MPV (fL)	6.53 ± 0.48	6.48 ± 0.31	6.42 ± 0.13	6.38 ± 0.19
P-LCR (%)	7.28 ± 3.27	7.04 ± 2.04	7.28 ± 1.05	6.10 ± 1.75

HCT – hematocrit, HGB – hemoglobin, MCH – mean corpuscular hemoglobin, MCHC – mean corpuscular hemoglobin concentration, MCV – mean corpuscular volume, MPV – mean platelet volume, PDW – platelet distribution width, PLT – platelets, P-LCR – platelet large cell ratio, RBC – red blood cells (erythrocytes), RDW – red cell distribution width, WBC – white blood cells (leukocytes), W-LCR – eosinophils, W-MCR – neutrophils + monocytes, W-SCR – lymphocytes, fL – femtoliter, g/dL – g/deciliter, pg – picogram. The values represent the mean ± SD and were analyzed by ANOVA. There were no significant differences between the responses to andiroba oil and the negative control, or among the doses of oil tested.

## Discussion

Toxicological and genotoxicity studies are required to assess the efficiency and safety of natural products used to treat diseases among Amazonian and other populations, whereas chemical analyses are important in determining the lipid profiles of these products. Assessment of potential genotoxicity is particularly important since such toxicity is considered to be fundamental in the development of diseases such as cancer (Ribeiro *et al.*, 2003). Lipids play an important role in the toxicity of natural oils, and investigation of the lipid composition of andiroba oil can provide insight into their contribution to the biological activity of this oil. In addition, the lipid profile of the original (natural) oil extracted from seeds and the profile of oil produced after fermentation of the seeds must be compared to determine whether there are significant differences between them.

Stearic, palmitic, oleic and linoleic acids can cause cellular apoptosis (Mu *et al.*, 2001; Lu *et al.*, 2003; Cury-Boaventura *et al.*, 2004), but nothing has been published about linked free fatty acids (FFA). Although there are major differences between FFA and esterified fatty acids, FFA are not neutral molecules and their biological activities are not the same as those of linked FFA. In this context, lipidomics provides a useful strategy for analyzing total and fractionated oils, with acidity being related to the FFA released during oil processing and extraction.

Since one of the pathways leading to apoptosis involves DNA degradation (Grivicich *et al.*, 2007) it is worth considering whether the concentrations of lipid components in andiroba oil are sufficient to trigger apoptosis by damaging genetic material. In this case, only FFA can cause damage and analysis of the unfractionated natural oil is required to provide a reference for comparison; the latter should be investigated first, followed by an analysis of oil fractions.

In this study, we used the comet and micronucleus tests, two validated genotoxicity bioassays, to screen andiroba oil for toxicity. These tests can identify damage to DNA and indicate its extent and gravity (Wong *et al.*, 2005; Asare *et al.*, 2011). The comet assay quantifies lesions to DNA in individual cells (Tice *et al.*, 2000; Collins, 2004), whereas the micronucleus test indicates chromosomal instability (Jagetia and Reddy, 2002). The PCE/NCE (polychromatic erythrocyte/normochromatic erythrocyte) ratio is another parameter that can be assessed during micronuclear analysis. The progression of erythroblasts from the PCE stage to NCE stage is an indicator of the acceleration or inhibition of erythropoiesis, with a decrease in the ratio indicating cytotoxicity (Venkatesh *et al.*, 2007).

As shown here, treatment of mice with andiroba oil did not damage the DNA of blood cells (comet assay), nor was there a significant increase in MN-PCE frequency or a decrease in the PCE/NCE ratio for any of the oil samples tested (micronucleus test). The latter finding indicated that there was no chromosomal structural and/or numerical damage in the erythroblasts of Swiss mice treated with andiroba oil and there was no cytotoxicity to bone marrow. Together, these findings indicate that andiroba oil is not genotoxic, cytotoxic or mutagenic. Similar results have been reported for other plant oils, such as oil from pequi (*Caryocar brasiliense*) (Roll MM, 2013, MSc dissertation, Universidade de Brasília, Brasília, DF, Brazil) and oil extracted from the fruit of *Litsea cubeba* (Luo *et al.*, 2005).

Since no genotoxicity was observed in this study, the apoptosis caused by fatty acids (stearic, palmitic, oleic and linoleic acids) may not originate from genetic damage, in a manner similar to that reported for limonoids, substances also found in andiroba oil that cause cell apoptosis via the mitochondrial route (Kikuchi *et al.*, 2011). Another possible explanation could be that the concentration of these fatty acids in andiroba oil is insufficient to trigger this type of cell death; this would mean that andiroba oil could be classified as a GRAS (Generally Recognized as Safe) product that is good enough to use in ointments.

The doses used in this study can be compared with those used by Costa-Silva *et al.* (2008) in acute tests with Wistar rats. These authors used a dose of andiroba oil (5 g/kg) that was much higher than that used here and also found no adverse effects in histological, biochemical and hematological analyses; they estimated the lethal dose (LD_50_) to be > 5 g/kg. Since mice, which have a low body mass, were used in the present study, we opted to follow the doses described in the OECD (1997) Guidelines 474 (mammalian erythrocyte micronucleus test) and 475 (mammalian bone marrow chromosome aberration test), these doses being 500, 1000 and 2000 mg/kg/day for 14 days.

There were no significant changes in body weight or in the absolute and relative weights for liver, spleen and the left and right kidneys after treatment. Costa-Silva *et al.* (2008) also noted that there were no significant alterations in Wistar rats treated for 14 days with andiroba oil at doses much higher than those administered here. Increases in plasma alanine aminotransferase (ALT) and in the relative and absolute liver weights are generally indicative of hepatic toxicity. The absence of such changes in the present study indicated that andiroba oil was not toxic to mice. There were also no significant changes in any of the blood parameters. Overall, these findings indicate that there were no significant alterations in any of the hematological, biochemical and morphological parameters investigated.

Since the doses of andiroba oil used in this study were similar to those used in phytotherapy, we conclude that the risk of toxicity from this oil when used in humans is very low. In addition, the DPPH test clearly demonstrated the antioxidant activity of all 21 samples of andiroba oil, although there was considerable variation among them. This antioxidant activity could protect DNA from oxidative damage. In an analysis of the chemical composition analysis of andiroba oil, Martinborough (2002) detected terpenes in the non-saponifiable portion of the oil; terpenes are believed to contribute to the antioxidant activity of the oil. However, Wu *et al.* (2013) indicated that the antioxidant activity of an essential oil can be attributed to components present in great quantity, although synergistic or antagonistic mechanisms may also be involved.

Based on the information available at the moment, it is not yet possible to attribute antioxidant activities to a specific compound in andiroba oil. Such identification would initially require separation of the oil components followed by individual analysis of the DPPH-reducing activity by thin layer chromatography (TLC) to compare the antioxidant activity of different classes of substances. The DPPH test was found to be useful for detecting regional as well as geographic variation in the antioxidant capacity of andiroba oil samples in the state of Pará.

Variations in antioxidant activity may be associated with climatic or environmental differences, or may reflect genetic variability and the diversity of collected specimens. In the state of Pará, the rains last from December to July, with peak rainfall from March to May (Moraes *et al.*, 2005). Among the samples analyzed, most collections occurred between March and July, thus covering much of the wet season. Consequently, the variation in antioxidant capacity probably did not reflect climatic differences. Tian *et al.* (2014) suggested that variation in the chemical composition and antioxidant capacity of the herb *Perilla frutescens* from which essential oil is extracted in China may be the result of genetic and/or environmental factors. Similar factors could account for the differences in the antioxidant capacity of andiroba oil. The DPPH test was particularly useful for screening andiroba oil that was sold diluted in mineral oil, as in the case of the Iana sample. Dilution in mineral oil reduces the oil's antioxidant capacity and attenuates the therapeutic properties such as acaricidal activity (Farias *et al.*, 2007, 2009), larvicidal action (Silva *et al.*, 2004) and insect repellent action (Miot *et al.*, 2004, 2011).

Comparison of the EC_50_ of the most active sample of andiroba oil, LAM 1 (EC_50_ = 43.56), and the sample used in most of the experiments described here, namely, SW3 (EC_50_ = 59.95), with samples from other studies, such as pequi oil (EC_50_ = 26.26) (Roll MM, 2013, MSc dissertation, Universidade de Brasília, Brasília, DF, Brazil) indicated that higher values were obtained with andiroba oil, *i.e.*, this oil had a lower antioxidant potential than pequi oil.

Overall, our data extend our knowledge of the risks of adverse toxic effects and the potential phytotherapeutic uses of andiroba oil. In the experimental conditions used here, andiroba oil was not hematotoxic, genotoxic, mutagenic or cytotoxic. On the contrary, the antioxidant activity of the oil would tend to protect cellular DNA from oxidative damage. In conclusion, andiroba oil, which is used in folk medicine among Amazonian populations, has a low risk of toxicity under the conditions in which it was tested here.

## Supplementary material

Figure S1 - Percentage inhibition of DPPH by Southeast (SE) samples 1-3. The points represent the mean ± SD of SE.

Figure S2 - Percentage inhibition of DPPH by Northeast (NE) samples 1-3. The points represent the mean ± SD of NE.

Figure S3 - Percentage inhibition of DPPH by Lower Amazon (LAM) samples 1-3. The points represent the mean ± SD of LAM.

Figure S4 - Percentage inhibition of DPPH by Marajó (MAR) samples 1-3. The points represent the mean ± SD of MAR.

Figure S5 - Percentage inhibition of DPPH by Metropolitan (MTR) samples 1-3. The points represent the mean ± SD of MTR.

Figure S6 - Percentage inhibition of DPPH by Southwest (SW) samples 1-6. The points represent the mean ± SD of SW.

Figure S7 - Percentage inhibition of DPPH by commercial samples of andiroba oil. The points represent the mean ± SD of commercial samples.
