# Supplementary material for: The lipidome, genotoxicity, hematotoxicity and antioxidant properties of andiroba oil from the Brazilian Amazon
Source: Genet Mol Biol. 2016 May 13;39(2):248–56. doi: 10.1590/1678-4685-GMB-2015-0098 (PMC4910557; doi:10.1590/1678-4685-GMB-2015-0098)
Supplement: Figure S2 [file 1415-4757-gmb-1678-4685-GMB-2015-0098-Suppl02.pdf]

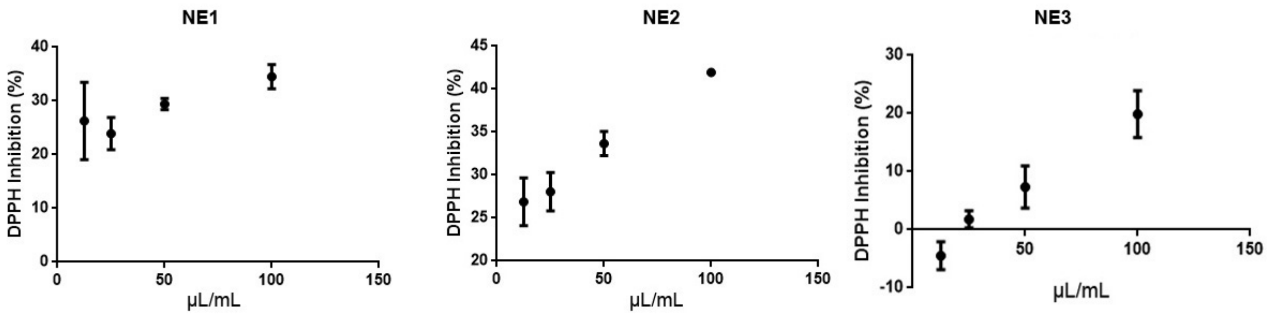

Figure S2 - Percentage inhibition of DPPH by Northeast (NE) samples 1-3. The points represent the mean ± SD of NE.
